# Offline Scaling of IoT Devices in IOTA Blockchain

**DOI:** 10.3390/s22041411

**Published:** 2022-02-12

**Authors:** Abhimanyu Rawat, Vanesa Daza, Matteo Signorini

**Affiliations:** 1 Department of Information and Communication Technologies, Universitat Pompeu Fabra, 08018 Barcelona, Spain; vanesa.daza@upf.edu; 2 NOKIA Bell Labs, 91620 Nozay, France; matteo.signorini@nokia-bell-labs.com

**Keywords:** IoT, blockchain, offline tangle, scaling, IOTA

## Abstract

An increased pattern of hidden Internet of Things (IoT) devices has been observed. Due to the increased number of security attacks, a large number of IoT devices are disappearing from the public internet. Operating blockchain operations in such ad hoc connectivity becomes challenging. However, multiple past studies have pointed towards IOTA Distributed Ledger Technology (DLT) that closely caters to offline blockchain use cases. However, there has been little to no empirical study or introduction to time bounds on transaction confirmation. Therefore, this study explains what provisions the existing IOTA blockchain has to accommodate the increased pattern of hidden IoT devices, and if IOTA is truly sufficient as a solution. In summary, we approach research questions by analyzing the studies that explore the trend of offline IoT devices and evaluating the relevance of offline blockchains, assessing the IOTA specification and codebase around offline transaction-making capabilities and pointing out some bounds that IOTA blockchain nodes must follow towards incoming transactions. Furthermore, we confirm by experimental runs that outside and within the tight time bounds transactions in offline Tangle can become stale and not get confirmed, and the effective time-bound can be even less. Realizing the need for a better offline blockchain scalability solution.

## 1. Introduction

IoT (Internet-of-Things) devices are inexpensive, lightweight, commodity hardware that are mostly used to interact with real-world or cyber-physical entities. They are tiny pieces of computational hardware, equipped with substantial storage and confined software that helps run the assigned task. Earlier in the 1990s, they were widely used in the industrial environment where physical tasks were automated. In current times, it has become easier to integrate IoT devices in more use cases such as smart-homes, automated vehicles, medicine, and hardware crypto wallets. With this development, the challenge to secure the huge data generated by IoT devices has become more pressing. Moving away from legacy solutions by alleviating the need for a trusted third party, the decentralized nature of blockchains can fit well with the IoT use cases [1]. Making data secure, available, immutable, while incurring low storage costs are some major advantages.

Blockchain promises decentralization, security, and access control across various application domains such as banking, decentralized finance (DeFi), supply chain, decentralized data management, and non-fungible tokens (NFTs). In technical terms, Blockchains are cryptographically secured DLT to achieve state-machine-replication [2], they are, by design, resistant to a Byzantine fault [3]. In a distributed ledger, the data can be thought of as a state which changes every time new data is applied and, through consensus protocols, other agents or peer-to-peer (P2P) nodes in the system, agree on the shared uniform state, i.e., a given majority have the same state or ordered view of the data. The most popular and prevalent type of DLT is Blockchain, which forms a series of cryptographically connected blocks [4] produced by other peer nodes called miners for a small fee. Inside blocks, a group of transactions can be packed and disseminated into the network and eventually agreed upon using various consensus algorithms. There are problems with block-based DLTs such as scalability [5], the fee (can be high at times), block production time, and Miner Extraction Value (MEV) [6]. This makes it difficult for blockchains to serve different use cases where such limitations are undesirable.

Outside block-based DLTs, other alternatives such as Direct Acyclic Graph (DAG) based DLTs have been introduced that somewhat alleviate the limitations of block-based blockchains such as validating large transactions than small or microtransactions, higher transaction fee, mining activity which is becoming more central than distributed [7,8,9,10]. DLTs are proven to be a game-changer in industry for example in cyber-physical systems where immutable data records hold utmost value. IOTA [10] is a notable example of this. In IOTA unlike blockchains like Bitcoin [11] or Ethereum [12], there are no miners and no fee to be paid in order to make a transaction thereby making access to the ledger easy.

**Figure 1:**
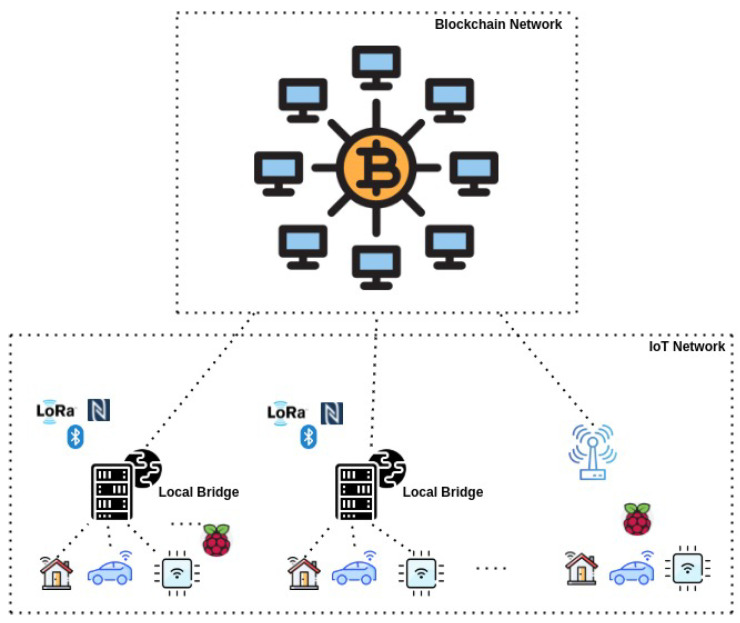
Traditional IoT blockchain architecture.

In traditional deployments, as seen in Figure 1, IoT devices use the blockchains via a common bridge or a local stub which actually serves as a point of contact on behalf of several IoT devices. Here, the trust assumption is moved from IoT devices to the common bridge, presenting a fake sense of decentralization and trust. Such deployments used to be the norm due to the technical difficulties [13] of putting blockchain applications on top of low-resource IoT devices. However, now this assumption has weakened with the advent of slightly more powerful IoT devices and less resource-demanding application software.

Lately, due to emerging cyber threats, research [14] has shown that IoT devices are kept hidden/isolated from the public internet which makes it difficult for them to use the blockchain infrastructure when securing the data they generate. It gives rise to the new type of use case for blockchains to consider, where some portion of the blockchain needs to be offline and periodically synchronized with the public part of the blockchain for the data to attain finality (irreversibly of blockchain state). In deployments such as supply-chain management or within an organization setup where the inter-IoT device communication can take place privately, having a periodical synchronization with public internet blockchain nodes will help achieve the required level of IoT device security and data finality requirements. In the rest of the document, the terms ledger and blockchain, and, finality and confirmation are used interchangeably.

This paper will investigate the IOTA blockchain which comes really close to solving the problem of offline blockchain while highlighting the further amendments required in order to meet the goals of offline blockchain. Our main contributions are:

1. Clearly formulating the problem of hidden IoT devices and their integration with blockchain.
2. Analyzing the existing blockchain (IOTA) which has some provision in place to address the problem of the offline Tangle.
3. Evaluating the feasibility of IOTA to accommodate the offline Tangle through code-level blockchain experiments.

The remainder of this paper is structured as follows: Section 2 describes the problem statement regarding the trend towards hidden IoT devices and blockchains. Section 3 gives a summary of related work on the subject of offline IoT devices and IOTA blockchain. Section 4 lays a foundation for the key aspects of IOTA blockchain which are relevant to this paper. Section 5 pinpoints the problems in IOTA offline scaling by exploring the relevant sections within the codebase. Section 6 provides an experimental analysis of IOTA nodes and discusses their impact on the offline Tangle use case. Section 7 briefly discusses the results and provides insight about potential future research direction. Section 8 concludes the paper.

## 2. Problem Statement

Since their inception, IoT devices have not received the timely upgrade when it comes to the evolution of hardware and software stack [15,16]. Even the communication protocols that came into existence in the late 1980s were ported to cater to the needs of the IoT devices much later; a similar pattern can be observed on other technical innovations as well [17]. The ignorance (https://www.forescout.com/wp-content/uploads/2016/06/ForeScout-Webtorials-IoT-Security-Survey-Results-June-2016.pdf, accessed on 21 December 2021) even continues on the upgrade and security cycles [18] where the lack of timely upgrades and a proactive approach towards security has exposed the IoT ecosystem to various types of attacks. With the advent of the internet and connectivity, IoT deployments can be easily accessed and managed via the public internet. Work by [14] showcases how easy it is to track the IoT devices in this way. Since most of the IoT devices use legacy communication protocols [19], it becomes easier to trace and attack them [14]. The visibility of IoT devices within the public internet poses a significant threat and attacks in the past have proven that this is a widely overlooked topic [20]. Research findings on the Mirai attack [21] explain in detail how a swarm of IoT devices spread around the world belonging to a variety of deployments and fragile network setup can be exploited to orchestrate a large-scale Distributed Denial of Service attack (DDoS) [22] attack. This can be one of the reasons why some organizations/states are actively making their IoT devices disappear from the public internet [14]. The following are two key problems that need to be addressed from observed trends. For our use case, we will study IOTA blockchain and see what provisions there are in IOTA and how such use case can be accommodated.

### 2.1. Hidden IoT Devices Using Blockchains

**Figure 2:**
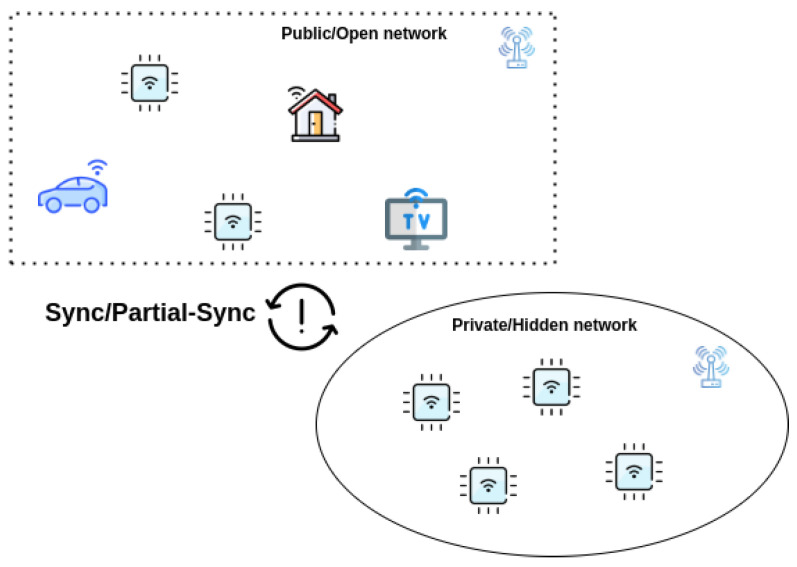
IoT devices under synchronous and partial-synchronous setup.

Public blockchains provide decentralization of data when the participating nodes remain synchronous with the blockchain network. Blockchain brings data/state immutability powered by a decentralized design. It becomes a critical use case to hide IoT devices so that some degree of a protective shield can be provided in terms of unreachability from the public internet (thus avoiding a wide range of attacks), however, at the same time, they must be a part of the public blockchain network to benefit from blockchain properties. As shown in Figure 2, there may exist network isolation between the IoT devices accessed via the public internet and IoT devices existing inside a private or hidden network. In this case, two types of network synchronization can be assumed.

1. **Synchronous**: In this type, the participating devices have full network connectivity with peer devices. In the IoT ecosystem, synchronous connectivity is unlikely since the network is also considered as one of the dearth resources.
2. **Partial-Synchronous**: In this type, the participating devices might have a random amount of network unreachability among them. In the IoT ecosystem, partial-synchronous connectivity is likely to be the case. Due to this, there is no reliable medium to exchange/synchronize the data among IoT devices, which can make blockchain operations impossible.

In a public blockchain, an inherent trust exists that there needs a majority of honest players in the network to have honest decisions. Having any data/state stored there brings a true sense of decentralization in some capacity. To have the data stored on the public blockchain, which is accessible via the public internet, IoT devices that are hidden/unreachable from the public internet need to periodically synchronize the data generated to the public blockchain.

Partial-Synchrony poses a more significant challenge since blockchains are designed to replicate data among P2P nodes in a real-time/synchronous way. There needs to be a mechanism by which some portion of the blockchain can continue expanding the blockchain until it is merged with the public portion of the blockchain periodically/with partial-synchronicity and thus attains finality.

### 2.2. Offline Blockchains for Isolated IoT Nodes

The topic of blockchain scaling is trending at present. The excessive volume of data, which directly impacts resource usage in terms of computation and storage, has affected the price or transaction fee and time/throughput in high-profile blockchains such as Ethereum and Bitcoin [5]. To counter this, multiple solutions have been put forward, both in terms of introducing novel consensus protocol blockchains such as Solana [23] and Avalanche [24], and scaling the base blockchain using techniques such as sidechains [25], Layer 2 (L2) [26], Plasma [27], and Lightning network [28]. Most of these solutions work in the domain of block-based ledger which is less suitable for IoT devices and does not support the case where a group of devices can continue blockchain operation under network isolation. In DAG-based ledgers, the scope of scaling is not well-defined [29] and its use cases have been difficult to comprehend as per any current and potential demand.

In this paper, we discuss the offline scaling aspects of DAG-based IOTA blockchain and how accommodating the real-world trend of IoT devices being taken offline will be a feature moving forward. As shown in Figure 2, the use of partial-synchronization of data between public blockchain and private networks where IoT devices are deployed can solve the problem described in Section 2.1. It is essential moving forward that blockchains are offering offline scaling along with other features as mentioned above in Section 2.2.

## 3. Related Work

Blockchains attained popularity when novel cryptocurrencies Bitcoin [11] and Ethereum [12] were announced. They are block-based, having a linear structure where several transactions are put inside a block. Through a consensus mechanism (Bitcoin uses PoW [30] while Ethereum uses PoW and Proof-of-Stake (PoS) [31]) the block is accepted by nodes present in the network. These blockchains use miners that solve cryptographical puzzles in PoW and hold a reasonable stake in PoS to grow the network; miners are computing nodes capable of carrying out very intensive computations. They are, however, a poor fit to accommodate the needs of the IoT ecosystem. Even if the difficulty of PoW is decreased, the whole block-based blockchains still do not meet the needs of IoT use cases, as described in Section 1.

Byteball blockchain [8] is permissionless and uses a DAG structure comprised of chained transactions instead of blocks. It uses witnesses, which are pre-selected trusted nodes to maintain the validity of transactions on the chain. A total of 12 witnesses are selected and they can be replaced with common nodes following a procedure. Growing the ledger privately and synchronizing it with the main chain is an issue in Byteball since it is not tolerant to the separate chain.

JHdag blockchain [32] is a permissionless blockchain where a single transaction is treated as a single block and uses PoW for Sybil protection as well as adding weight (reputation) to a block that later can be translated to a particular type of block, for example, a normal transaction or consensus related transaction. It has miners in its network which add blocks following rules such as pointing to the miner’s previous block, pointing to a previous milestone from the main chain under the longest chain rule, and pointing to another miner’s block. Such conditions would make it quite complex to accommodate the private/offline ledger use cases.

In the work of Karlsson et al. [33], a fault-tolerant permissioned blockchain has been proposed where, in order to let the IoT blockchain grow under network faults assumption there is a support blockchain and powerful relay nodes. The solution is novel but relies heavily on support blockchain and relay nodes that drive both complexity and cost.

More theoretical and exploratory work [34] has been put forward by Alsboui et al. The issue of offline resiliency is very briefly described with quite a narrow description of what it entails and what the solution would look like. Here no data and studies have referred to why it is a problem worth solving other than IoT attributes. They have pointed out that IOTA has the capability but have not described the underlying details of whether it will actually work or not. Our paper explains it more holistically and showcases it via experimental runs using currently used IOTA mainnet node software.

A hybrid blockchain model based on top of IOTA is proposed by Hellani et al. [35]. It comprises edge servers and a base blockchain sitting somewhere in the cloud. Having such layered infrastructure poses more complexity and drives up the cost of infrastructure. The proposed architecture makes an assumption that the IOTA Tangle is capable of working under an offline Tangle scenario but no bounds such as amount of offline time or unconfirmed transactions rate have been described. A similar assumption can also be found in other papers [36,37,38,39], they are completely agnostic to the underlying functioning and what bounds nodes need to abide by when they refer to IOTA capabilities for offline transactions. As seen in Table 1, different attributes are mentioned including offline compatibility, to the best of our knowledge, there does not exist any study or reference on the offline compatibility before this one. Our work attempts to provide relevant bounds on offline compatibility for IOTA blockchain using a practical estimation backed by real data on offline transactions trying to attain finality and trade-offs along the way if IOTA blockchain is used as a solution.

## 4. IOTA Background

**Figure 3:**
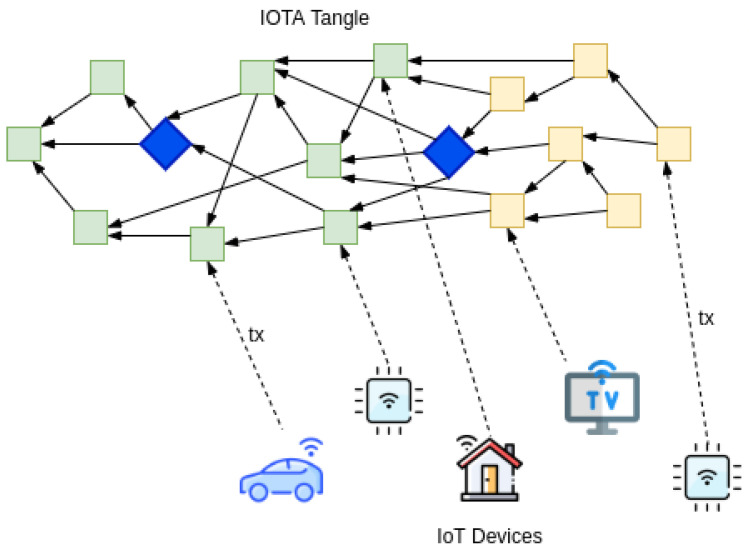
IoT devices interacting with IOTA ledger.

IOTA ledger, unlike other block-based DLTs, is more suitable to the needs of IoT or cyber-physical devices such as no transaction fee, high scalability, and throughput. As shown in Figure 3, IoT devices get access to the IOTA ledger, and the transactions (tx) sent by IoT devices are added to the growing ledger. After a certain amount of time, the transactions attain finality. Any IoT device running the IOTA node software is able to add and gossip transactions to its peers. IOTA software follows a light design to lower the resource consumption so that a wide range of IoT devices can directly run the node software and do not depend on any third-party service.

**Figure 4:**
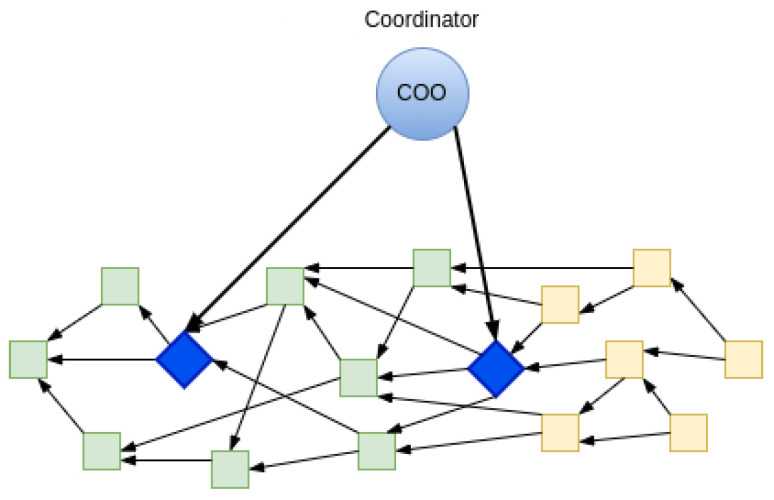
Transactions in IOTA ledger confirmed by Coordinator.

Here is a brief overview of how the IOTA blockchain works. The structure of IOTA DLT is called a Tangle [10]. Nodes in the IOTA network share Tangle and the incoming transactions are validated or added as per the consensus rules. As shown in Figure 4, instead of a group of mining nodes packing transactions inside the block, every new transaction can be a part of the growing tree of transactions in an acyclic way by referencing some existing transaction and thus forming a graph. A new incoming transaction validates the previous ones (2-8) and helps grow the IOTA network. In this way, many transactions can be validated in parallel providing high throughput and scalability. A special node called Coordinator (COO) helps transactions present in the IOTA ledger attain finality, more on Coordinator mentioned below. Since there is no fee for producing a transaction, to protect the ledger from Denial-of-service (DoS) attacks or spamming transactions, a small amount of proof-of-work (PoW) is required which does not take part in the consensus but provides a weight for the transaction to be considered valid.

Not all the nodes in the IOTA network have exactly the same view of the Tangle, i.e., some transactions nodes choose not to include as well [10]. Generally, such transactions either have a low reputation or are not necessary for the node to add them unless a new transaction references them. There are some major components that make up the IOTA Tangle such as Mana (Available online: https://blog.iota.org/explaining-mana-in-iota-6f636690b916/, accessed on 21 December 2021) (a reputation system in IOTA 2.0, future release), Coordinator, Fast Probabilistic Consensus (FPC) [40], and, Tip Selection. In the rest of this section, we will introduce some key building blocks and underlying processes that make IOTA function.

### 4.1. Coordinator

In the present IOTA implementation (Available online: https://github.com/gohornet/hornet, accessed on 21 December 2021), every node comes with a hardcoded address of a special node called Coordinator. The Coordinator acts as a central entity that helps IOTA transactions to achieve finality by releasing the milestones at regular intervals. In IOTA, transactions per second (TPS) are different from confirmed transactions per second (CTPS). The former is when a node accepts the new incoming valid transaction and adds it to its Tangle and also broadcasts it to its peer nodes. But the transactions achieve finality (which is a way of making sure that the state of the ledger is then irreversible) using special transactions called milestones. At present, the IOTA foundation controls the Coordinator node and in future releases, i.e. IOTA 2.0 or Coordicide, plans to get rid of it.

### 4.2. Milestones

**Figure 5:**
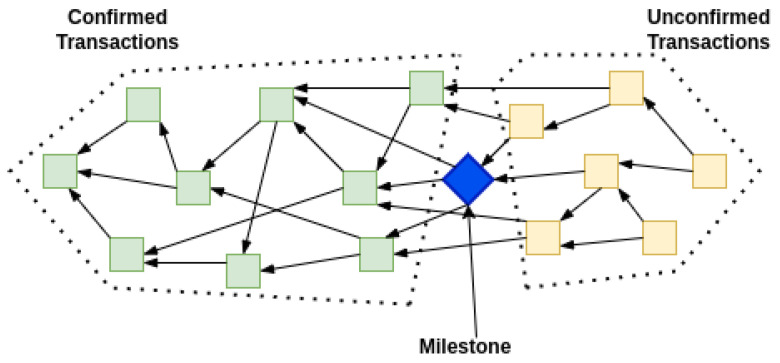
Milestone transaction confirming other transactions.

Milestones are generated by the Coordinator node following some rules slightly different than when making a normal transaction. A milestone refers to a number of transactions, and past transactions, directly or indirectly referred by them, become confirmed or final [10]. So, all the transactions reachable via a milestone are considered as final or irreversible. On average there is a 30-s threshold to trigger the milestone if it is not generated automatically. As shown in Figure 5, the milestone transaction (in blue) directly or indirectly confirms the previous transaction (in green) in a Tangle. While the transactions in yellow are not yet confirmed.

It used to be the case that a transaction validates two or more transactions but post white-flag (Available online: https://iota.cafe/t/conflict-white-flag-mitigate-conflict-spamming-by-ignoring-conflicts/233 accessed on 21 December 2021) update a transaction refers to two or more transactions which may or may not be valid. Just because a milestone validates a transaction does not mean all past transactions in that cone are valid, instead, invalid transactions are instructed to be ignored.

### 4.3. Offline Tangle

There may exist a case where a large number of IOTA nodes go unreachable/offline from other public nodes as well as from the Coordinator while still making transactions and thus growing the sub-Tangle (sub-DAG). Such use case can be termed the offline Tangle. It adds the capability for the subset of nodes to continue operations uninterrupted with the caveat that while they are offline/isolated, they will not be able to reach finality, i.e., transactions referred by a milestone directly or indirectly. Such transactions form a cone (a sub-DAG) composed of transactions done offline and, ideally, the cone should be announced eventually to the public nodes in order to attain finality.

**Figure 6:**
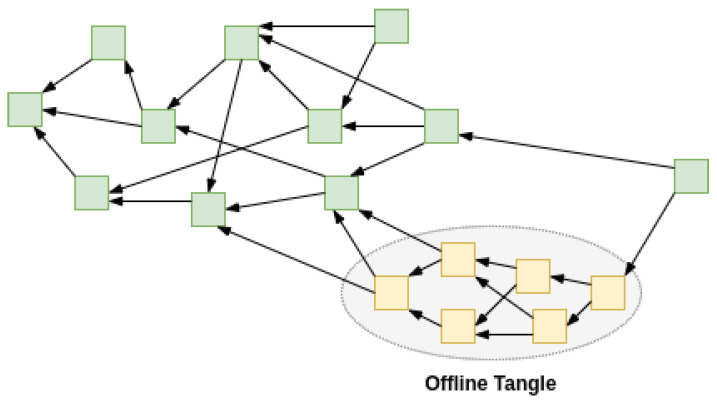
Offline Tangle synchronizing transactions.

As shown in Figure 6, a group of transactions (in yellow on grey background) were performed offline and made available to the main Tangle and referenced by another transaction from the main Tangle. The offline transactions have now peered across the IOTA network.

### 4.4. Solidification

A given message/transaction is considered solid if all the transactions in its past are present or can be reached by the node. When a transaction is received by a node, it checks if its parents exist or not. If not, they are then requested based on an attribute which is unique to every message called message ID. This is a recursive process that continues until the full history of the original transaction is available. With solidification, it is easy to check if the transaction and its history are valid or not. Usually, a node broadcasts a solidification request with the message ID of the transaction to its neighbors and if they have it, then they send it back to the requesting node. The solidification process works in the following two ways:

1. **Solidification using a Milestone**: to make the solidification process faster the node gives a milestone index to the neighboring nodes and all the transactions referred via the given milestone are sent to the node.
2. **Solidification using message ID**: This is mostly used when the node already has the milestone transaction reference. The message IDs are broadcasted in parallel fashion, for example, for a transaction any unavailable parents are requested in parallel.

## 5. Offline Scaling Analysis in IOTA

To tackle the problem mentioned in Section 2 there needs to be a mechanism to scale the blockchain to accommodate the isolated transactions which are valid in nature. It may look like a partition tolerance use case since in DAGs such partitions are quite common for a short period of time [41]. But when such partitions are desirable to accommodate some use cases where a large number of transactions are voluntarily isolated/put offline, it makes it a scalability issue because there should be a mechanism that ensures those transactions will be included in the majority public nodes view. This section, we will discuss the existing technology used in IOTA protocol and ask to what degree it can solve the isolated/offline blockchain problem.

### 5.1. Tip Selection Woes

In the IOTA blockchain, transactions are appended to the DAG/Tangle validating 2–8 transactions (tips) selected from a pool of transactions called the tip pool. To be considered as a potential tip, an incoming transaction needs to go through a scoring system, based on which it is decided whether or not a given transaction qualifies to be included or dropped from the tip pool. Newly arrived transactions are given three types of scores:

1. **Lazy**: Transaction which is not considered to be a part of the tip pool.
2. **Semi-Lazy:** Transaction which might be confirmed but without being part of the main tip pool. Currently, only Non-Lazy tip pool is considered (Available online: https://github.com/gohornet/hornet/blob/764e4ee25995b1557129277a97dea244b923513e/plugins/restapi/v2/node.go#L79 accessed on 21 December 2021).
3. **Non-Lazy**: Transactions that are eligible to be included in the tip pool.

Lazy transactions are cones of unconfirmed transactions that exist too far in the past to be included, i.e., a lot of time has elapsed since any of these transactions were directly or indirectly referred by the regular milestone. Consequentially, it will be too much work for the node to validate all the transactions present in the cone, and there is no guarantee that at the end of the validation process all the transactions found in that cone will be valid. In a way, it is an avoidance technique where nodes discard the cone where there is a possibility of validation work getting wasted if transactions are not valid. Algorithmically, IOTA nodes have set the depth of about 15 milestones deep from the latest milestone present at the fully synchronized node. If the difference between the latest milestone and the oldest cone root index is higher than 15, then straight away that transaction is discarded.

Meanwhile, the Non-Lazy and Semi-Lazy transactions still have a chance to be confirmed by a node.

These classifications serve as some preventive measures against the resource exhaustion attacks, for example, denial of service (DoS) and finality delay, but also result in resisting the merge of an offline Tangle with the public Tangle present in publicly reachable nodes.

At the time of writing this paper, the average number of messages per second on IOTA is 15. The average rate of milestone generation is around 12 s. The total number of transactions between a milestone is around 180 transactions (Available online: https://explorer.iota.org/mainnet accessed on 21 December 2021).

In order to avoid the offline Tangle not being included in the main Tangle, the offline Tangle should broadcast its cone before almost every 180 × 15 = 2700 transactions or 15 (max depth limit) × 12 seconds/milestone = 180 seconds.

Since the number of transactions may vary with different use cases, time is a better unit of measurement to avoid the offline Tangle being discarded.

#### 5.1.1. Solidification Effect on Offline Tangle

Some nodes that are working offline want to merge their sub-Tangle with the main Tangle. Such nodes might run into a situation where the time limit under which it is safer to broadcast the message is further shortened if the solidification requests take a lot of time.

A number of transactions take place in a sub-set of nodes that are unreachable from the public blockchain nodes. Now, the solidification process is dependent on those offline node(s) once such nodes decide to put the transactions online. Since only offline node(s) have the transaction history of the offline Tangle then such nodes act as a single point of service. So, one or a few nodes having offline transactions that can be broadcasted to the public blockchain nodes can adversely affect the time calculation which is previously assumed in Section 5.1. If the solidification takes longer than usual then there is a risk that the sub-Tangle will not merge with the main Tangle at all.

Let the rate of solidification requests sent by a node be α upon receiving a transaction that is not solid yet, and the height of the cone which remained offline be *H*. So, the time to solid becomes α·H

In parallel, if the normal operations at the node and network such as milestone generation are working normally then the effective time to broadcast the offline Tangle can be computed as:

(1) effectivetime=15(maxdepthlimit)·12seconds/milestone−α·H.

Hence, Equation (1) provides a true bound on the time limit under which it is suitable to let the offline Tangle merge with the Tangle present in the public IOTA nodes.

## 6. Experiments

To showcase the issues discussed on IOTA scaling as mentioned in Section 5, experiments are developed to closely observe and analyze the problems using an official IOTA private Tangle implementation framework (Available online: https://github.com/iotaledger/one-click-tangle accessed on 21 December 2021). The experiments are entirely based on the official IOTA Github repository that uses the currently operational IOTA node software (version Hornet) which is a part of the Chrysalis update (1.5). This section will discuss the setup specifications and the experimental observations.

### 6.1. Setup Specifications

Using the official private Tangle implementation framework provided by IOTA, we have orchestrated the DLT network comprising the following:

- **2 Nodes**: They are standard IOTA nodes present in the network which receives the transactions from other nodes, validate them, attach them to their local ledger, and finally broadcast them to their peers. Both of them are added as their neighboring nodes.
- **2 Spammers**: They are similar to the nodes mentioned above but specially designed to generate a lot of traffic in the form of spam transactions. Spamming the IOTA network will help grow the ledger/the Tangle and closely mimic a real-world behavior.
- **1 Coordinator**: Special node provisioned to release regular milestones to provide finality to the network. As defined in Section 4.1.
- **3 Networks**: Offline Tangle 1 and Offline Tangle 2 networks to make a partition between the subgraph with the Coordinator and subgraph without a Coordinator. Private Tangle network that connects the main nodes to each other in order the synchronize the transactions.

**Figure 7:**
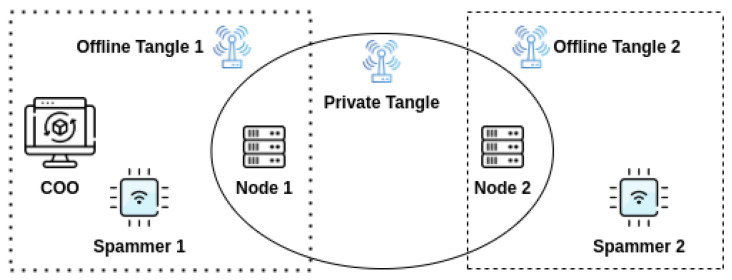
Experimental setup of IOTA network.

As shown in Figure 7, Node 1 and Node 2 are a part of the Private Tangle network that enables them to synchronize their transactions with each other. At the same time, Node 1 is a part of the Offline Tangle 1 network which connects Node 1 to the Coordinator and Spammer 1. If Node 1 is disconnected from the Private Tangle network then it still receives transactions from Spammer 1 and milestones from the COO. The same is the case with Node 2, which is also connected to the Offline Tangle 2 to receive the transactions from Spammer 2.

### 6.2. Platform Specifications

The official IOTA private Tangle setup lets a user orchestrate any IOTA network use case on docker containers. For this experiment, the 11th generation i7-1165G7 @2.80GHz x 8 core processor and 512 GB SSD powered by Ubuntu 20.04.3 LTS is used. Docker version 20.10.11 along with docker-compose 1.23.2 are used.

### 6.3. Experimental Analysis

We ran the use cases which allowed us to study the issues mentioned in Section 5.1 and Section 5.1.1. We made minor changes to the configuration of IOTA private tangle implementation to accommodate the given issue. Changes include adding another additional normal node, spammer node, and private networks to orchestrate the offline use case. The experimental runs are open (Available online: https://github.com/WiSeCom-UPF/one-click-tangle/blob/chrysalis/hornet-private-net/README_Research.md accessed on 2 February 2022) for everyone to run and observe similar results. The experimental setup is a one-click solution with the following manual adjustment to orchestrate the particular use cases under observation:

- **Node 1 Network Disconnect**: The goal of the exercise is to observe the behavior once the public blockchain nodes are unreachable or a group of IoT nodes are isolated. Public IOTA nodes including the COO, will become unreachable by issuing the network disconnect command. This way a group of IOTA nodes will be isolated.

#### 6.3.1. Tip Selection Woes

The issue of tip selection woes concerns the possibility that a subset of nodes or sub-tangle network in IOTA is working in an isolated manner, i.e. transactions are unreachable to the Coordinator for a while. This use case is quite realistic since a vital characteristic of IoT devices includes their ad-hoc nature.

To observe the intended behavior in experimental analysis, we kept the belowMaxDepth variable low enough, i.e., 8. belowMaxDepth is a configurable parameter which is the numerical difference of milestone a node allows in an incoming transaction. Any value greater than belowMaxDepth will make the transaction not selected for being part of the tip pool. The time to milestone interval is set at 10 s, it is the interval between two successive milestones issued by the COO. This way, every 80-s period of non-synchronization among Node 1 and Node 2, will lead to a very high chance that upon synchronization post 80-s period, the offline Tangle at Node 2 will become stale and unreachable from the new set of transactions. From the experiment runs, we observed that the old offline part of the Tangle was detached entirely from the new one.

**Figure 8:**
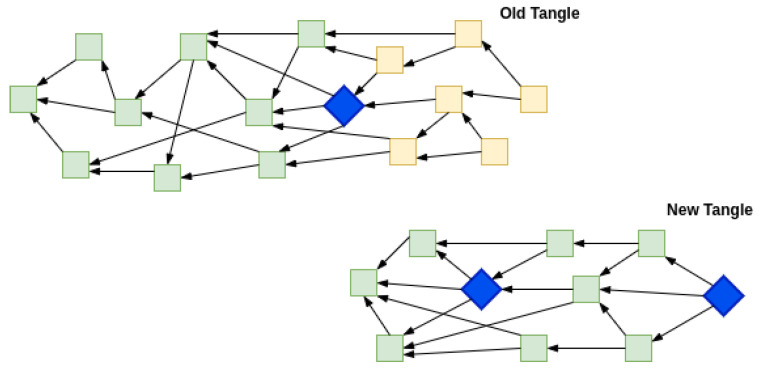
Tangle splitting virtually into two.

**Figure 9:**
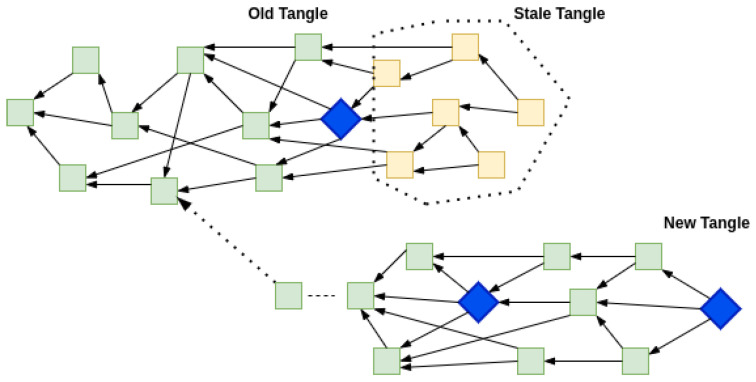
Logical representation of Tangle split.

The IOTA visualizer can not update the old data if there is a significant gap between the new and old transactions. Due to this, two visualizations can be drawn from the experiment. As shown in Figure 8, the old Tangle after the milestone (in blue) was disconnected from the Coordinator and after the time mentioned in Equation (1) when the Coordinator was again reachable then the unconfirmed transactions (in yellow) became disconnected from the new Tangle. Visually, it can be imagined as a Tangle disconnect but it is more virtual than logical. As shown in Figure 9, a confirmed transaction (in green) in a new Tangle if traced back to its history will be referring to a transaction before the last seen milestone in the old Tangle. The trend shown in experimental runs develops as per the expected behavior described in Section 5.1. It is the group/cone of unconfirmed transactions (shown in yellow) that disconnected from the Tangle as it has been classified as having Lazy tips followed by older transactions.

#### 6.3.2. Solidification Effects

From successive runs, we noticed that the adequate time as mentioned in Equation 1 is indeed a good estimation to broadcast the transactions of offline Tangle. It was due to the fact that there is only Node 2 which was exposed to Node 1 and Node 2 had all the offline transactions which were to be solidified by Node 1. The present solidification algorithm of IOTA works in a breadth-first fashion, so if the missing cone has height N then it will take at least N separate requests to solidify the missing cone.

There is another means of solidification where instead of message IDs per level, the node asks for transactions belonging to a particular milestone. This is a highly efficient way to solidify incoming transactions. However, it is not Tangle in our use case since no milestone got issued as the transactions of the subjected Tangle were not reachable from the Coordinator node.

#### 6.3.3. Caveats of Tip Selection

There is something very interesting which we have noted from the experiments. Even with a short period of isolation from the main Tangle where the Coordinator can reach the newly generated transactions, a significant number of transactions fail to be referenced by the Coordinator, in other words, they fail to attain finality. There is no Tangle split like the one mentioned in Section 6.3.1, but it leaves a lot of re-doing proof-of-work to reproduce the transactions which were not confirmed.

The reason behind such behavior is the fact that due to the large accumulation of unconfirmed tips, it becomes very competitive once a node after some time unloads the transactions to the Coordinator. At the Coordinator, the probability of getting the tips that covered all potential subgraphs in a Tangle becomes very low. This best-effort system by the Coordinator is agnostic towards the underlying Tangle structure, because at the fast-moving pace, figuring out the tips that cover most of the Tangle is still an open problem.

**Figure 10:**
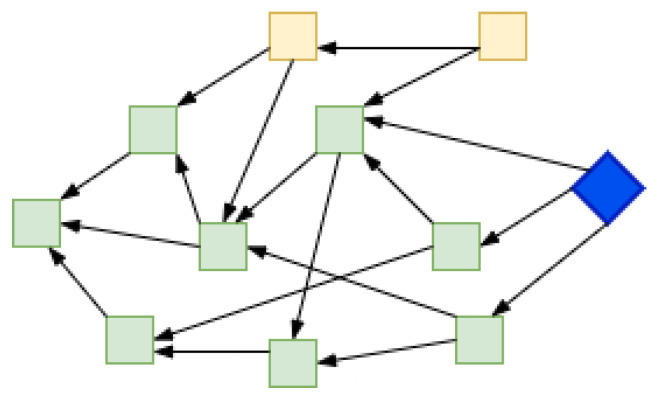
Transactions unreachable from Milestone.

As shown in Figure 10, the green transactions are confirmed by milestone transaction (in blue) but the yellow transactions are not reached via the Milestone. The yellow transactions were not picked by any future milestone and thus remained unconfirmed.

If a node is in sync with the Coordinator without downtime then it is rare that any transaction is left unconfirmed. Having little downtime is expected in the case of IoT devices but losing a significant number of transactions because they are unconfirmed raises a serious issue.

## 7. Discussion

Fundamental properties of IoT devices that affect blockchain adoption include their limited computation power and network reachability. It is an absolute requirement for a blockchain solution to be resilient to network discontinuations for an IoT deployment. Considering the cyberattacks IoT devices face from the public internet, the fundamental property of network resiliency becomes a required feature. In a mission-critical setup, such as cyber-physical deployment which usually has its intranet, can leverage a lot from an ideal offline blockchain. In this case, the IoT devices can carry their operations normally within an enclaved/isolated network and rapidly synchronize their data to the public blockchain or a larger trustful group of nodes.

We show in this paper that the existing provisions in the IOTA blockchain provide quite limited aid to offline devices that want to synchronize their data to the public blockchain nodes. IoT devices may be unreachable by the public either internet voluntarily or involuntarily, but in both cases, the solution in IOTA blockchain seems far behind what it should ideally be. One cannot control what configuration parameters (apart from the default suggested by IOTA) a random public node uses to help finalize the offline transaction. With current default configurations, it takes just a few minutes for an IOTA public node to accommodate offline transactions. Beyond this time period, the offline nodes can risk losing the opportunity for their offline transactions to reach finality. When offline nodes broadcast their transactions to the public peer nodes, then the broadcasted transaction competes against other transactions for the tip selection process. Since the tip selection process is strictly based on the transactions which are not made too far in the past, voids/narrow the offline blockchain objective. Even with offline transactions broadcasted within the time bounds, a significant proportion of those transactions still might not get confirmed. To our understanding, provisions in IOTA make the blockchain resilient to a very small network outage, as defined in Equation (1), while retaining the trade-off of putting a portion of transactions through the reattachment process. As shown in Table 2, the results pointed by our study fills a gap between theory and hypothesis made by past studies that IOTA accommodates the offline blockchain use case. Our study is the first of a kind that investigates different aspects of offline blockchain from IOTA’s point of view. Issues investigated in our study will also lay a foundation for future blockchains that aims to support offline functionality. Our study also points out the impact of data availability on time bounds as noted in Equation (1) and convergence of transactions abiding the time bounds. The offline blockchain is a far more valuable and technically challenging use case, especially considering the projections that in the next 10 years, the IoT market will grow exponentially.

There may arise cybersecurity concerns around the role network might play, for example, eclipsing a network of IOTA nodes that they become unreachable from the COO. In other blockchains such as Bitcoin and Ethereum, a node can be eclipsed by malicious nodes and fed incorrect information, leading to further attacks such as double-spending. For an IOTA node, the consequence of such an attack will be the force reattachment process, which drains resources of IoT devices. The more time a group of IOTA nodes are out of synchronization from the COO, the more damage the attack will incur. Such an attack can be orchestrated without even breaking any existing IOTA protocol rules, sufficient eclipsing of the IOTA nodes and a network unreachability of the COO can substantially affect the normal operations.

The IOTA solution might not be complete, but it is certainly a step in that direction. For IOTA blockchain, there is a conservative estimate that any node must have some trust-based assumption that an incoming cone of transactions are valid ones and work done to validate them will not go to waste. For this, they use the depth (number difference) of milestones in the past as a better estimate, but there is a potential for other trust-based metrics. Regarding future work, we would suggest either a protocol-level packet change or a special transaction, or some PoW based difficulty that lets public nodes trust the cone of transactions. An empirical study regarding the cybersecurity aspect of offline IOTA blockchain can be a valuable future contribution.

## 8. Conclusions

With the increased number of IoT devices taken offline to protect them from cyber attacks, there is an emerging need for blockchains that support offline scalability. This study experimentally analyzed the IOTA specification around offline blockchain functioning. Use cases such as the effects of tip selection algorithm on offline transactions, solidification effect, and partial synchrony were performed under the offline Tangle scenario, where a subset of nodes was unreachable from the Coordinator (public IOTA nodes). Results showed splitting of the Tangle, unconfirmed transactions, and less adequate time to synchronize with the public ledger/Tangle. These results are significant for both IoT device operators and blockchains that claim or aspire to provide offline blockchain functionality. Scaling the offline Tangle in its current shape and structure seems quite challenging since it would require protocol-level severe changes to accommodate a more extended offline Tangle period with a higher transaction confirmation rate.

More efficient scaling techniques could encourage the IoT industry towards blockchain-based solutions without worrying about putting extra resources and outside protocol mechanisms in place.

## Figures and Tables

**Table 1:** Table to compare attributes of past studies.

Blockchain	Type	Structure	Finality	Offline Compatibility
Byteball	permissionless	transaction based DAG	trusted 3rd party	not defined
JHdag	permissionless	block based DAG	trusted 3rd party	not defined
IOTA	permissionless	transaction DAG based	trusted 3rd party	not defined
PoW based (Bitcoin & Ethereum)	permissionless	block based chain	PoW miners	not supported
Karlsson et al.	permissioned	hybrid block based	trusted 3rd party	not defined

**Table 2:** Our results compared to the past studies. Offline functionality specifications are neither defined in DAG-based Bytebal, JHdag, and IOTA blockchains, nor in past studies and solutions that depend on IOTA’s offline blockchain functionality.

Studies Issues	Time Bounds	Data Availability	Convergence within Bounds
Byteball	No	No	No
JHdag	No	No	No
IOTA	No	No	No
Other studies [33,34,35,36,37,38,39]	No	No	No
Our Study	yes	yes	Yes

## Data Availability

https://github.com/WiSeCom-UPF/one-click-tangle/blob/chrysalis/hornet-private-net/README_Research.md (accessed on 2 February 2022).
